# Schizophrenia: the announcement of the diagnosis

**DOI:** 10.1192/j.eurpsy.2023.2238

**Published:** 2023-07-19

**Authors:** F. Azraf, M. Chtibi, F. Laboudi, A. Ouanass

**Affiliations:** Arrazi Hospital, Faculty of Medicine and Pharmacy of Rabat, Sale, Morocco

## Abstract

**Introduction:**

For many years, the question of the announcement of the diagnosis in psychiatry has been controversial. It is the starting point of a management allowing access to psychoeducation and to the patient’s recovery. In the case of schizophrenia, the cognitive impairment and the stigmatizing nature of the pathology jeopardize the announcement of the diagnosis. However, recommendations and legislation emphasize the need to inform the patient about his or her pathology. In Morocco, the law n° 131-13 of February 19, 2015 relating to the practice of medicine has made information about the diagnosis to patients an obligation for doctors and a right for patients

**Objectives:**

The interest of our work is to try to evaluate the current state of this practice, its ethics and its representations among psychiatrists.

**Methods:**

This is a descriptive study on the announcement of the diagnosis of schizophrenia in a population of psychiatrists. The data collection was carried out by a questionnaire including: Socio-demographic and professional data, opinion on practice and training concerning diagnostic announcement in psychiatry, physicians’ representations concerning announcement: frequency, opinion on the importance of this practice.

**Results:**

31 participants responded to our questionnaire. More than 9 out of 10 participants would not benefit from training on diagnostic announcement. Only 22.6% of physicians reported being very or somewhat familiar with medical information laws and their content regarding the regulation of diagnostic announcement. All participants considered schizophrenia to be the most difficult pathology to announce, followed by personality disorders and bipolar disorder. 74.2 of the participants considered it “rather” or “completely” essential to inform the patient of his or her psychiatric diagnosis. 77.4% of the participants considered it necessary to announce the diagnosis of schizophrenia and 80.7 often or systematically announce this diagnosis. Three situations considered appropriate to announce a diagnosis of schizophrenia: 74.2% announce it in general when the patient or the family asks for information about the diagnosis, 42% advise the patient when he/she mentions schizophrenia on his/her own. The patient’s functional inability to understand the diagnosis (77.4%) and the fear of negative clinical and therapeutic repercussions (41.9 and 38.7 respectively) were reported to deter physicians from making the announcement. More than half of the participants (64.5%) thought that the announcement of the diagnosis improved therapeutic compliance. Conversely, 35.5% considered that the announcement had no impact on therapeutic compliance.

**Conclusions:**

The announcement of the diagnosis of schizophrenia remains today a complex and evolving subject. Even if great progress has been made to inform patients as well as possible, practices remain disparate from one doctor to another and this information is not well traced.

**Disclosure of Interest:**

None Declared

